# Context‐dependent functional dispersion across similar ranges of trait space covered by intertidal rocky shore communities

**DOI:** 10.1002/ece3.2762

**Published:** 2017-02-17

**Authors:** Nelson Valdivia, Viviana Segovia‐Rivera, Eliseo Fica, César C. Bonta, Moisés A. Aguilera, Bernardo R. Broitman

**Affiliations:** ^1^ Instituto de Ciencias Marinas y LimnológicasFacultad de CienciasUniversidad Austral de Chile, Campus Isla TejaValdiviaChile; ^2^ Centro FONDAP de Investigación de Dinámicas de Ecosistemas Marinos de Altas Latitudes (IDEAL)ValdiviaChile; ^3^ Departamento de Biología MarinaFacultad de Ciencias del MarUniversidad Católica del NorteCoquimboChile; ^4^ Centro de Estudios Avanzados en Zonas Áridas (CEAZA)Universidad Católica del NorteCoquimboChile

**Keywords:** community assembly, desiccation, environmental filtering, functional beta‐diversity, functional traits, marine, mesoscale, seasonal

## Abstract

Functional diversity is intimately linked with community assembly processes, but its large‐scale patterns of variation are often not well understood. Here, we investigated the spatiotemporal changes in multiple trait dimensions (“trait space”) along vertical intertidal environmental stress gradients and across a landscape scale. We predicted that the range of the trait space covered by local assemblages (i.e., functional richness) and the dispersion in trait abundances (i.e., functional dispersion) should increase from high‐ to low‐intertidal elevations, due to the decreasing influence of environmental filtering. The abundance of macrobenthic algae and invertebrates was estimated at four rocky shores spanning ca. 200 km of the coast over a 36‐month period. Functional richness and dispersion were contrasted against matrix‐swap models to remove any confounding effect of species richness on functional diversity. Random‐slope models showed that functional richness and dispersion significantly increased from high‐ to low‐intertidal heights, demonstrating that under harsh environmental conditions, the assemblages comprised similar abundances of functionally similar species (i.e., trait convergence), while that under milder conditions, the assemblages encompassed differing abundances of functionally dissimilar species (i.e., trait divergence). According to the Akaike information criteria, the relationship between local environmental stress and functional richness was persistent across sites and sampling times, while functional dispersion varied significantly. Environmental filtering therefore has persistent effects on the range of trait space covered by these assemblages, but context‐dependent effects on the abundances of trait combinations within such range. Our results further suggest that natural and/or anthropogenic factors might have significant effects on the relative abundance of functional traits, despite that no trait addition or extinction is detected.

## 1 Introduction

The study of functional diversity can reveal the mechanisms that underpin the assembly of communities (Hooper et al., 2005; Mouchet, Villéger, Mason, & Mouillot, 2010). Functional diversity expresses the diversity of functional traits, which are those components of an organism's phenotype that influence its response to environmental stress and its effects on ecosystem properties (Hooper et al., 2005; Petchey & Gaston, 2006). Accordingly, processes related to niche differences among species, such as niche complementarity, are intimately linked with variations in functional diversity (Mason, de Bello, Mouillot, Pavoine, & Dray, 2013). For instance, steep environmental gradients, like in wetlands and intertidal rocky shores (Berthelsen, Hewitt, & Taylor, 2015; Mudrak et al., 2016), can drive spatial shifts in composition from functionally similar species due to the selection of traits adapted to the local abiotic and biotic conditions (i.e., trait convergence), to functionally dissimilar species that occupy different niches (i.e., trait divergence; Boersma et al., 2016; de Bello, Leps, & Sebastia, 2006). Functional divergence, in turn, allows the coexistence of functionally dissimilar organisms through niche complementarity (Mason, de Bello, et al., 2013). Assessing how different facets of functional diversity vary from convergence to divergence and the spatiotemporal persistence of these patterns could improve our ability to unravel the mechanisms underlying community assembly.

Ecosystems characterized by strong environmental stress gradients provide an opportunity to understand how communities assemble through the spatiotemporal variation in functional diversity. Environmental filtering occurs when environmental stress exceeds the physiological limits of tolerances of individuals in local populations (Somero, 2010), which are selected for subsets of organisms with similar functional traits that allow them to persist in a given area (Leibold et al., 2004). Such trait convergence can be represented by reduced functional diversity within the assemblage (e.g., Fukami, Bezemer, Mortimer, & van der Putten, 2005). As environmental stress decreases, reduced similarity and increased niche differences among competing individuals allow coexistence, leading to a stronger influence of niche complementarity on community assembly (MacArthur & Levins, 1967; Mason, de Bello, et al., 2013). This functional divergence leads to increased functional diversity during community assembly (e.g., Mudrak et al., 2016). As a consequence, species‐rich communities should show reduced functional diversity in areas where environmental stress is high compared to areas where stress is low.

Functional diversity is a multifaceted concept that can be represented on a multidimensional space where the axes are functional traits (recently reviewed in Carmona, de Bello, Mason, & Leps, 2016). In such *trait space*, functional richness (Villéger, Mason, & Mouillot, 2008) represents the range occupied by a given community and can be estimated as the number of functional trait combinations. Functional dispersion (Laliberté & Legendre, 2010) is estimated as the mean distance of all species to the weighted centroid of the community in the trait space, being equivalent to the multivariate dispersion (Anderson, Ellingsen, & McArdle, 2006). When the influence of species richness on these indices is statistically removed (Mason, Lanoiselee, Mouillot, Wilson, & Argillier, 2008), functional richness and dispersion are “pure” estimators of the occupied range of functional space and the dispersion in trait combination abundances, respectively (Mason et al., 2012). Interestingly, functional richness and functional dispersion can show differing spatiotemporal patterns of variation. Environmental stress would not be strong enough to filter out functional roles, but it would be enough to generate significant differences in the number of individuals within functional trait combinations (Boersma et al., 2016). In temperate regions, for instance, the seasonal variation in environmental conditions and recruitment intensity leads to significant changes in the abundance of marine seaweeds (Cavanaugh, Siegel, Reed, & Dennison, 2011; Valdivia, Gollety, Migne, Davoult, & Molis, 2012). Then, these environmentally driven effects would result in a significant variation in multivariate trait dispersion (i.e., varying functional dispersion) despite that all trait combinations are present under the full range of environmental conditions (i.e., similar functional richness).

Intertidal rocky shore communities represent an ideal habitat to investigate how functional diversity varies along environmental stress gradients, because of the strong vertical gradients observed within a few meters for abiotic factors such as temperature, desiccation, irradiance, and osmotic potential (Gómez & Huovinen, 2011; Menge & Branch, 2001; Raffaelli & Hawkins, 1996; Stephenson & Stephenson, 1949). In these systems, competition for settlement space increases as the abiotic environmental stress decreases from high to low vertical elevations, and biotic interactions such as predation and grazing prevent competitive exclusion in the low‐intertidal zone (e.g., Connell, 1961; Donahue, Desharnais, Robles, & Arriola, 2011; Paine, 1974). Then, niche complementarity should take a larger role in determining species occurrences and abundances, allowing more species to coexist in the less stressful environment (MacArthur & Levins, 1967). Regarding the horizontal variation in community structure, local and mesoscale factors such as productivity, wave exposure, and recruitment variability modulate species abundances and occurrences to lesser extent (Navarrete, Wieters, Broitman, & Castilla, 2005; Roughgarden, Gaines, & Possingham, 1988; Valdivia, Aguilera, Navarrete, & Broitman, 2015). For instance, high wave‐exposure can hamper the grazing activity of low‐intertidal gastropods and sea urchins, allowing for competitive exclusion by algae (e.g., Hawkins & Hartnoll, 1983; Underwood & Jernakoff, 1981, 1984). Environmental filters and biotic interactions have therefore interdependent selective effects on trait combinations and thus on functional diversity. Despite the large body of literature arising from the study of intertidal rocky shore communities, less work has been carried out on how different facets of functional diversity vary at different spatiotemporal scales in these ecosystems (but see Berthelsen et al., 2015).

In this work, we analyzed a dataset of 68 intertidal macrobenthic species (macroalgae and both mobile and sessile invertebrates) to test the hypothesis that functional richness and dispersion increase from high‐ to low‐intertidal elevations, because of decreasing effects of environmental filtering on trait combination’ occurrences and abundances along the vertical stress gradient. We further assessed the persistence of local patterns of functional richness and dispersion over multiple sites (covering ca. 200 km) and times (seasonal samplings over three years) in a region showing a mesoscale structure in ecological subsidies such as chlorophyll‐*a* concentration and invertebrate recruitment (e.g., Van Holt et al., 2012).

## 2 Materials and Methods

### 2.1 Study region and environmental conditions

**Figure 1:**
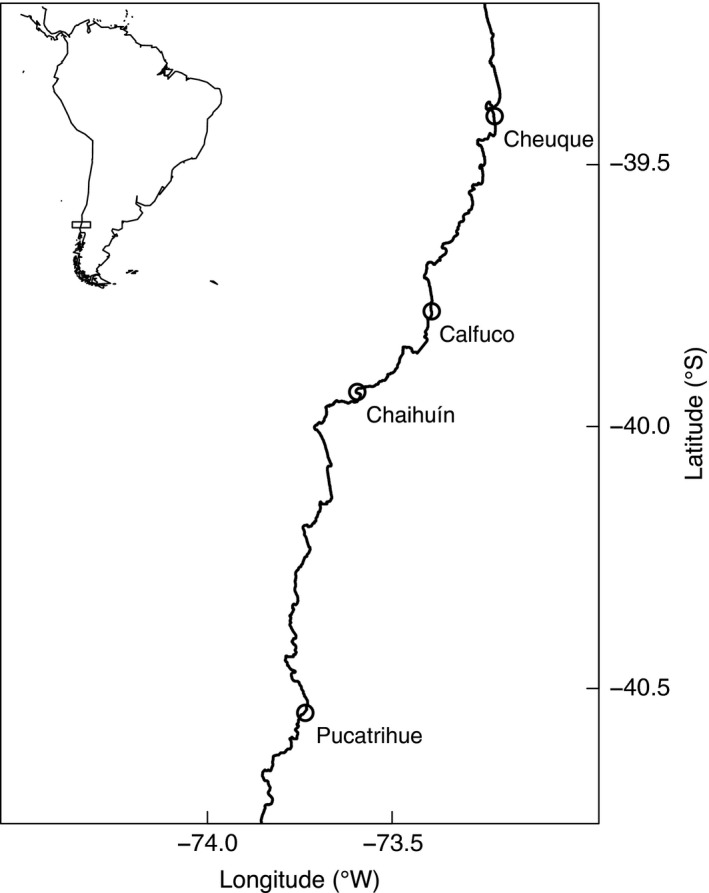
Location of sampling sites along southeast Pacific shores. The insert indicates the geographic area covered by our sampling program

Between June 2012 and June 2015, four intertidal shores were sampled every 6 months along ca. 200 km of the shore (Figure 1; 39.5°–40.5°S). The shores were Cheuque, Calfuco, Chaihuín, and Pucatrihue (Figure 1). The sites are representative of wave‐exposed southeastern Pacific shores in a region characterized by a significant mesoscale variation (i.e., between 10s and 100s of km) in chlorophyll‐*a* concentration, related to freshwater inputs and apparently to runoff from forestry and agricultural activities (Van Holt et al., 2012). In addition, a seasonal upwelling center is located in Punta Galera (Letelier, Pizarro, & Nunez, 2009), few km to the south of Chaihuín (Figure 1). Calfuco is an open‐access shore, where no regulation on fishermen activity is enforced. On the other hand, Cheuque, Chaihuín, and Pucatrihue are management and exploitation areas for benthic resources where the exploitation of intertidal macroalgae and invertebrates by small‐scale artisanal fishermen is regulated (Gelcich et al., 2010). During the last decades, the intensification and southward migration of the Southeast Pacific Subtropical Anticyclone have led to a strong cooling of coastal sea surface temperatures in the region of the southeastern Pacific located to the south of 33°S (Ancapichún & Garcés‐Vargas, 2015).

Monthly data (2003–2014) of sea surface temperature and chlorophyll‐*a* concentration (SST and Chl‐*a*, respectively) were analyzed to characterize the environmental conditions in the four sampling sites. Satellite‐derived MODIS data were downloaded from http://oceancolor.gsfc.nasa.gov/. Air temperature data would have been desirable to better characterize the environmental conditions to which intertidal organisms are exposed. However, air temperature data for southeastern Pacific shores are available at too coarse resolution (1°), which would have not fitted to our study.

Across these shores, intertidal habitats are dominated by sessile species such as the acorn barnacles *Jehlius cirratus* and *Notochthamalus scabrosus* (high intertidal), the purple mussel *Perumytilus purpuratus* and the red corticated algae *Mazzaella laminarioides* (mid intertidal), and large brown kelps such as *Lessonia spicata*,* Durvillaea antarctica*, and *Macrocystis pyrifera* (low intertidal; Moreno, 2001; Gómez & Huovinen, 2011). Grazers such as the keyhole *Fissurella picta*,* Chiton granosus*, and *Scurria zebrina* can have significant effects on the structure and zonation patterns of intertidal macroalgae (Moreno & Jaramillo, 1983). Smaller grazers, such as the pulmonate gastropod *Siphonaria lessoni,* littorinids, and amphipods, are abundant in this region (Moreno, 2001)—these grazing species are mainly associated with patches of the numerically dominant *P. purpuratus* and can have a strong top‐down effect on the assembly of sessile communities (Jara & Moreno, 1984; Tejada‐Martinez, López, Bonta, Sepúlveda, & Valdivia, 2016).

### 2.2 Sampling

At each site and sampling time, we estimated species abundances on ten 0.25 m^2^ plots located along ca. 20‐m alongshore transects. Transects were replicated at three intertidal elevations, low‐, mid‐, and high‐intertidal zones. On each bench, we used perennial marine species occurring highest on the shore as indicators of the upper intertidal boundary. Studies on wave‐exposed shores conducted elsewhere show that their upper distribution limit on different sites represents a summary of the local wave regime across multiple sites (Harley & Helmuth, 2003). The barnacle *J. cirratus* is the sessile and perennial species occurring highest on these shores. Once the upper boundary was determined on each shore, we divided the intertidal range into three zones of roughly equal vertical extent (i.e., high, mid, and low zones). Plots were haphazardly positioned along each elevation zone, but positions were restricted to flat and gently sloping surfaces lacking deep crevices and tide pools.

All seaweeds and invertebrates (>5 mm) occurring on each plot were identified in situ. Organisms were classified to the lowest possible taxonomic level (usually species). For each plot, we used a 50 × 50 cm frame, divided into 25 equal fields with monofilament line, to estimate species abundances. Sessile and mobile species abundances were estimated as percentage covers (1% resolution) and densities (ind./m^2^), respectively. This same protocol has been used in several studies of benthic diversity along Chile and elsewhere (Broitman et al., 2011; Bulleri et al., 2012). In order to simultaneously analyze sessile and mobile species, percentage cover data were transformed to proportions of the maximum observed for each taxon across the shores. In this way, all cover and density data ranged between 0 (the absence of species in a given quadrat) and 1 (maximum value across all quadrats; Caro, Navarrete, & Castilla, 2010; Valdivia et al., 2015). All species abundance estimations were conducted during diurnal low‐tide hours.

Each taxon was categorized according to four functional categories: hatching mode, adult body size, growth type, and trophic type (Table 1). The information of functional traits was obtained mostly from the published literature (Brusca & Brusca, 2003; Chen, Soong, & Chen, 2008; Ríos, Noziglia, & Guzmán, 1987; Hines, 1986; Kay & Emlet, 2002; Levin & Bridges, 1995; Moran, 1997; Narvaez, Navarrete, Largier, & Vargas, 2006; Reynoso‐Granados, Monsalvo‐Spencer, Serviere‐Zaragoza, & Del Proo, 2007; Saelzer, Quintana, & Quiñones, 1986; Venegas, Ortiz, Olguin, & Navarrete, 2000) and experts’ personal communication. In some cases, genus‐ and family‐level information was used when more specific information was unavailable. We then calculated functional richness and functional dispersion (referred to as FRic and FDis hereafter, respectively) as estimators of functional diversity (Laliberté & Legendre, 2010; Villéger et al., 2008). As our functional traits were defined as categorical variables, FRic was estimated as the number of unique trait combinations. To estimate functional FDis, we used principal coordinate analysis (PCoA) axes as the new quantitative traits (Villéger et al., 2008). PCoA axes were computed from a square root‐corrected Gowen dissimilarity matrix among the species (Laliberté & Legendre, 2010). Then, FDis was calculated from the mean distance of species to the centroid of the resulting multivariate trait space—each distance was weighted by the relative abundance of the corresponding species (Laliberté & Legendre, 2010).

**Table 1:** Functional trait categories analyzed in this study

Hatching mode	Adult body size
PD Planktonic development	S *<*1 cm
NPD Nonplanktonic development	M 1–10 cm
SP Spore	L 10–100 cm
	XL 100–1,000 cm
	XXL >1,000 cm

Growth type	Trophic type
E encrusting	A autotroph
B bushy	P predator
F filamentous	S suspension feeder
M massive	G grazer
L Foliose	D deposit feeder

The spatial or temporal patterns of FRic and FDis can be largely influenced by collinear patterns of species richness. In order to account for this collinearity, we estimated the standardized effect size of FRic (i.e., SESFRic) and FDis (i.e., SESFDis) according to Mason et al. (2008) and Mason, de Bello, et al. (2013). By this method, the observed FRic and FDis values are contrasted to those expected from matrix‐swap models (Manly & Sanderson, 2002) that randomize species’ occurrences (for FRic) and abundances within observations (for FDis). The SES estimators were then calculated according to Gotelli and McCabe (2002). Null model analyses used 10,000 randomizations. In this way, SESFRic and SESFDis were “pure” estimators of functional richness and the dispersion in trait combination abundances, respectively (Mason et al., 2012). Negative (positive) values of both estimators indicate that functional richness and dispersion are lower (greater) than expected at random.

### 2.3 Statistical analysis

Separate random‐slope mixed models were fitted to SESFRic and SESFDis in order to test our prediction. In these models, intertidal height (high, mid, and low intertidal) was included as fixed factor, and sampling site and time (nested in site) were included as random factors. In addition, the autocorrelation of errors, due to repeated measurements over time of each site, was specified as an autoregressive structure (AR1). Model diagnostics were checked by visually inspecting autocorrelation of residuals and fitted‐versus‐residual plots. We compared the second‐order Akaike information criterion corrected for small sample sizes (AICc) in order to select the more parsimonious model that accounted for the variation in SESFRic and SESFDis (Burnham & Anderson, 2004). A delta AICc > 2 was used as decision rule for selecting two competing models (Burnham & Anderson, 2004). The random‐slope models allowed us to determine whether the intercepts and slopes of the effects of intertidal height on both estimators of functional diversity varied between sites and between sampling times.

We used similarity routines (SIMPER) to determine the trait combinations that accounted for most of the differences between intertidal heights, sites, and times. SIMPER was based on a Bray‐Curtis dissimilarity matrix. Environmental SST and Chl‐*a* data were visualized by means of multivariate redundancy analyses with site as the grouping factor. All analyses were conducted in the R programming environment version 3.3.0 (R Core Team, 2016). We used the libraries FD, mgcv, picante, nlme, MuMIn, vegan, and sciplot.

## 3 Results

**Figure 2:**
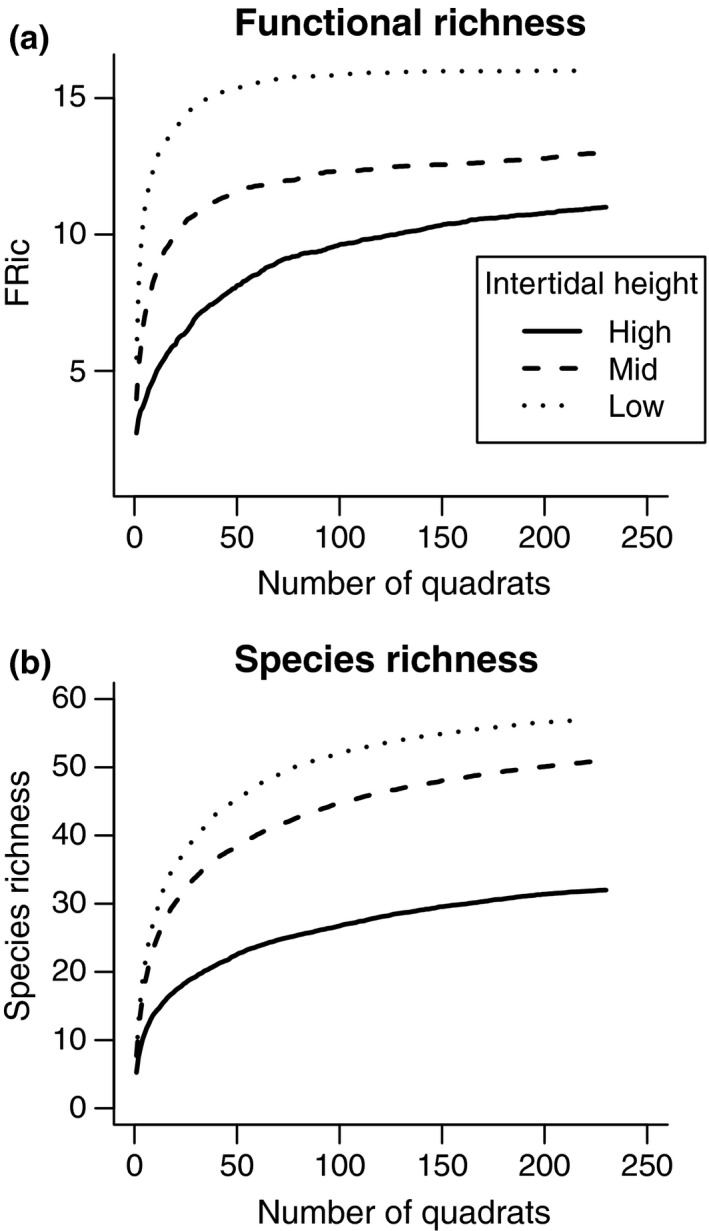
Accumulation curves for functional richness (FRic) and species richness for each level of the vertical intertidal environmental stress gradient (high, mid, and low heights). FRic was the number of unique functional trait combinations and species richness the number of taxonomic units

Our sampling protocol provided representative estimations of the number of unique trait combinations (i.e., functional richness, FRic) and taxonomic units (i.e., species richness; Figure 2). Asymptotic values of FRic ranged from ca. 10 to 15 unique combinations for high‐ and low‐intertidal heights, respectively (Figure 2a). Species richness ranged from ca. 30 in the high intertidal to 55 in the low intertidal (Figure 2b). According to these collinear patterns between FRic and species richness, the estimation of standardized effect sizes was appropriate.

The analysis of nearshore environmental conditions revealed that the sites located to the north of the study region were characterized by, in average, higher SST and Chl‐*a* concentrations than the southern sites (Fig. S1). This result agrees with previously published analyses showing higher Chl‐*a* concentrations to the north of the region (Van Holt et al., 2012).

**Figure 3:**
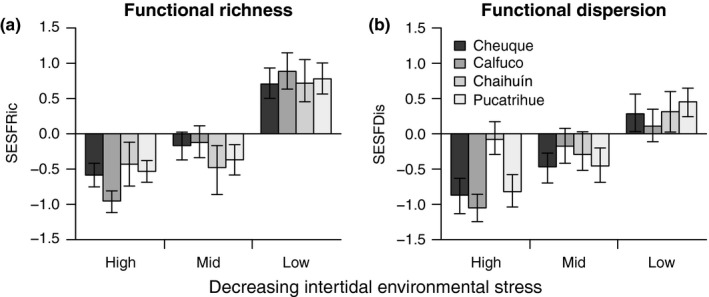
Patterns of variation of the standardized effect sizes of functional richness and functional dispersion (SESFRic and SESFDis, respectively) related to the vertical intertidal environmental stress gradient and sampling sites. SESFRic was calculated after randomizing the species occurrences according to a matrix‐swap null model. SESFDis was obtained by means of a null model in which abundances were randomized across species, but within sampling plots (Mason, Wiser, et al., 2013). Values are given as means ± confidence intervals

**Figure 4:**
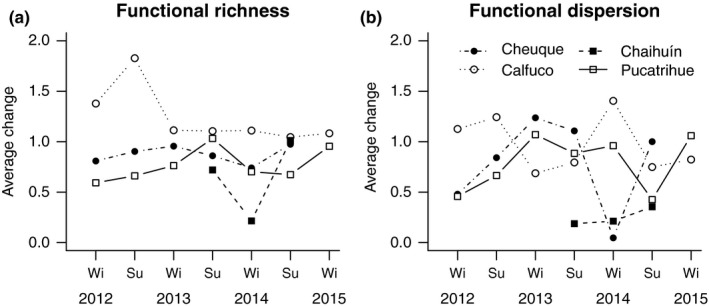
Coefficients of random‐slope models for the effects of the vertical intertidal environmental stress gradient (average change) on SESFRic and SESFDis at four rocky shore sites and over 3 years. SESFRic and SESFDis were calculated according to Mason, Wiser, et al. (2013). For each estimator of functional diversity, the coefficients represent the net change from high‐ to low‐intertidal heights at each site and sampling time. The coefficients were calculated from the full models in Table 2. Wi and Su are austral winter and summer, respectively

Standardized effect sizes for functional richness and functional dispersion showed positive trends with decreasing environmental stress (Figure 3; SESFRic and SESFDis, respectively). At high‐intertidal elevations, SESFRic and SESFDis exhibited values lower than those expected at random, hinting for trait convergence. At mid elevations, both estimators of functional diversity showed either lower or similar values than expected at random (Figure 3). At low‐intertidal elevations, most sites displayed values of functional diversity that where greater than expected at random, which suggests trait divergence. SESFRic showed relatively similar patterns of variation among sites, excepting for the mid‐intertidal height, where two sites displayed mean SESFRic values close to zero (Figure 3a). SESFDis showed a larger amount of geographic variation, with a balance between functionally similar and dissimilar species at every environmental stress level (Figure 3b). The results of random‐slope models supported these observations, as intertidal height was always selected in the most parsimonious models of the variation in SESFRic and SESFDis (Table 2). In addition, the fixed‐effects coefficients of intertidal height were all positive (Figure 4), supporting the prediction that functional richness and dispersion increase with decreasing environmental stress.

**Table 2:** Summary of results of random‐slope model selection for the standardized effect sizes of functional richness and functional dispersion (SESFRic and SESFDis, respectively)

Model	Fixed	Random	*df*	logLik	AICc	Δ*i*	ω_*i*_
Functional richness (SESFRic)							
1	*H*	—	5	−860	1,730	0.00	0.7428
3	*H*	*H*|*S* + *H*|*T*(*S*)	17	−849	1,733	2.28	0.2379
2	*H*	*H*|*S*	11	−858	1,738	7.30	0.0193
Null	—	—	3	−952	1,911	180.44	<0.0001
Functional dispersion (SESFDis)							
3	*H*	*H*|*S* + *H*|*T*(*S*)	17	−908	1,852	0.00	0.9975
1	*H*	—	5	−927	1,864	12.20	0.0022
2	*H*	*H*|*S*	11	−923	1,868	16.18	0.0003
Null	—	—	3	−976	1,958	106.14	<0.0001

For each competing model, fixed (intertidal height = *H*) and random (site = *S* and time = *T*(*S*)) components, in addition to the degrees of freedom (*df*) and log‐likelihood function (logLik), are given. Model were selected according to second‐order Akaike information criterion, corrected for small sampling sizes (AICc), Δ*i*, which is the difference between the AICc of each model and the smaller AICc, and Akaike weights (ω_*i*_).

The fixed‐effects coefficients from the random‐slope models showed differing spatiotemporal patterns between SESFRic and SESFDis (Figure 4). Average effects of the vertical intertidal stress gradient on SESFRic remained relatively constant over time and sites, excepting for the summer 2013 in Calfuco and winter 2014 in Chaihuín (Figure 4a). For SESFDis, average effect sizes of intertidal height presented a comparatively high variation over time, which was dependent on the sampling site (Figure 4b). Accordingly, the most parsimonious model for SESFRic selected via AIC*c* retained only the fixed effect of intertidal height—for SESFDis, the selected model retained intertidal height and the random effects of site and time (Table 2). Therefore, the results of model selection procedure indicated that the relationship between local environmental stress and functional dispersion, but not functional richness, significantly varied over space and time.

**Figure 5:**
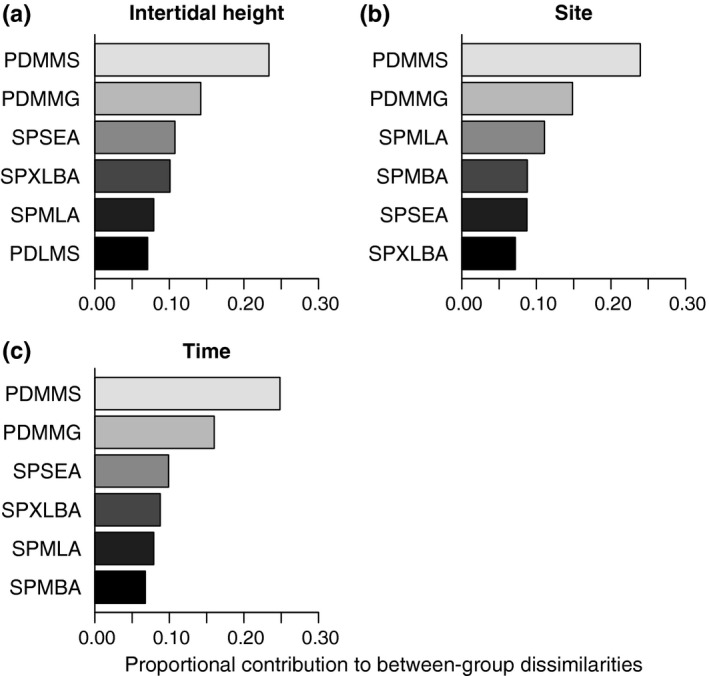
Proportional contribution of unique functional trait combinations to between‐group Bray‐Curtis dissimilarities for each source of variation (intertidal height, site, and time). Each contribution was calculated as the mean contribution of all of the average pairwise dissimilarities for each factor. A cutoff of 70% of contribution was used. Trait combination codes are as in Table 1—for example, PDMMS, the trait combination contributing most to differences between heights, sites, and times, is the combination of planktonic developer (PD), mid‐sized (M), massive (M), and suspension‐feeding (S) organisms

Medium‐sized, massive, and suspension feeder planktonic developers were the organisms that accounted for most of the Bray‐Curtis dissimilarities between intertidal heights, between sites, and between sampling times (Figure 5). Grazer species were also important to explain the between‐group differences for the three factors (Figure 5). Small‐sized encrusting macroalgae were ranked in third place to account for the variation across the vertical intertidal stress gradient and sampling times (Figure 5a,c). For the differences between sites, medium‐sized foliose algae accounted for ca. 11% of dissimilarities (Figure 5b).

## 4 Discussion

In this study, we showed that different facets of functional diversity had differing patterns of spatiotemporal variation. A significant increase in functional diversity along the vertical intertidal stress gradient was consistent across varying environmental conditions (in terms of SST and Chlo‐*a* concentration) and over more than 200 km and 36 months when the range of trait space covered by the local assemblages (functional richness) was analyzed. When the relative abundances of individuals within trait combinations were incorporated in the analyses (functional dispersion), the slopes between functional diversity and decreasing environmental stress significantly varied between sampling sites and times. Importantly, these effects were independent of the spatiotemporal variation in species richness. Suspension‐feeding planktonic developers and encrusting and foliose autotrophs were the functional traits that accounted for most of the spatiotemporal variation in abundances. Therefore, local environmental stress gradients might have persistent effects on the range of trait space covered by the local communities, but context‐dependent effects on the trait combination abundance distribution. Our results stress the relevance of analyzing multiple facets of functional diversity to understand the response of community assembly mechanisms to the environmental variability.

In agreement with our hypothesis, these results provide evidence for trait convergence and divergence along a steep environmental gradient over very small spatial scales. The negative values of the standardized effect sizes for functional richness (SESFRic) indicated that high‐intertidal assemblages encompassed functionally similar organisms, which were characterized by small body sizes. In general, trait convergence has been usually explained as the result of environmental filters that allow a subset of organisms with similar trait combinations to colonize and survive in patchy environments (e.g., Leibold et al., 2004). In central Chile, small‐sized barnacles that share similar environmental tolerances in the high intertidal show no evidence for competitive advantages of one species over the other, suggesting that ecological equivalence can be common at this height of the shore (Shinen & Navarrete, 2010, 2014)—complementarity in other traits, such as filtration rates (e.g., Valdivia et al., 2009), should be further analyzed to assess niche partitioning in this model system. At the low‐intertidal fringe, we observed that SESFRic was greater than expected at random, indicating the occurrence of species with dissimilar functional traits and an expanded coverage of the trait space (Pavoine & Bonsall, 2011). Predation of dominant competitors such as medium‐sized mussels by shallow subtidal seastars and muricid gastropods could facilitate the establishment of subordinate species (Moreno, 2001; Navarrete & Castilla, 2003; Paine, 1966) with different traits and thus increase functional divergence. In other systems, grazing limpets have been shown to exert a firm control on the upper limit of low‐intertidal macroalgae, preventing substrate monopolization (Boaventura et al., 2002). Our results further suggest that the role of niche complementarity in determining species occurrences and abundances increases with decreasing local environmental stress (MacArthur & Levins, 1967; Mason, de Bello, et al., 2013). Testing this hypothesis will require field‐based manipulations of functional diversity (e.g., Aguilera & Navarrete, 2012; Griffin, Mendez, Johnson, Jenkins, & Foggo, 2009) across intertidal heights, which will foster our understanding of the degree to which niche complementarity influences the assembly of these communities.

Contrarily to functional richness, the patterns of standardized effect sizes of functional dispersion (SESFDis) showed significant variations between sites and between seasonal samplings. This spatiotemporal variation in functional dispersion would be the result of changing environmental conditions strong enough to influence species abundances, but not enough to produce local or temporal extinctions. The higher Chl‐*a* concentrations observed at the northern sites could imply a higher food supply for planktotrophic larvae and adult suspension feeders, leading to greater invertebrate colonization rates and abundances (e.g., Menge, Gouhier, Hacker, Chan, & Nielsen, 2015). In our study region, the sites exposed to higher Chl‐*a* concentrations also exhibit higher invertebrate recruitment rates (Van Holt et al., 2012; N. Valdivia, unpblish. data), which would explain the spatial variation in the abundance of planktonic developers and suspension feeders observed in this study. The sites with greater Chl‐*a* concentrations also showed warmer waters. Along Chilean shores, and particularly in northern and central regions, the links between nearshore oceanography and coastal ecological processes are strongly related to upwelling activity (e.g., Lagos, Castilla, & Broitman, 2008; Navarrete et al., 2005). In central Chile, for example, upwelling‐related nutrient inputs are associated with greater abundances of corticated algae, but lower abundances of ephemeral algae and grazing effects (Nielsen & Navarrete, 2004). In southern central Chile, the upwelling center locked to Punta Galera (south of Chaihuín, Figure 1) might well encompass a source of environmental variability for coastal communities. Indeed, the sites in our study showing the higher Chl‐*a* concentrations and SST are located in the lee of this upwelling center (Pinochet‐Velásquez, 2015). Small bays downwind from upwelling centers can be areas where surface waters are retained close to the shore, resulting in warmer waters (Piñones, Castilla, Guiñez, & Largier, 2007) and greater larval retention (e.g., Morgan & Fisher, 2010). Nevertheless, the activity of the upwelling at Punta Galera is highly limited to spring and summer (Letelier et al., 2009), and the SST difference between the upwelling and shadow areas is relatively small (Pinochet‐Velásquez, 2015). Further studies are needed to determine the effects of this upwelling center on the spatial variability of functional traits in this region. Upwelling effects on species abundances and several relevant ecological processes have been shown worldwide, with stronger effects localized at areas characterized by more intermittent upwelling activity (e.g., Menge & Menge, 2013). In this way, nearshore oceanographic conditions can have strong effects on the abundance of functional trait related with, at least, dispersal potential and trophic strategies.

In addition to abiotic environmental factors, anthropogenic impacts should be considered to explain the patterns of functional trait variation along Chilean shores. Human harvesting in these shores has significantly altered the assemblages at the landscape scale. In particular, the overexploitation of keystone predators, such as muricid gastropods, has severely reduced their abundances on the shore, despite small‐sized individuals can be still observed (Moreno, 2001). This could increase the occurrences of small‐sized carnivore species (e.g., *Acanthina monodon*; Moreno, 2001), leading to trait convergence in terms of similarity in body size. A similar pattern has been observed for keyhole limpets, and small‐sized herbivores seem to dominate the assemblage of grazers in these shores (Tejada‐Martinez et al., 2016). Moreover, high‐intertidal macroalgae, such as *Pyropia orbicularis*, and low‐intertidal kelps, such as *D. antarctica* and *L. spicata*, are also under severe harvesting (Moreno, 2001). The region under study also shows spatial heterogeneity in these human impacts. Three of the sites are managed, while one is under an open‐access regime. Between the enforced sites, there are also important differences in the consequences of management for the natural communities, depending on differences among stakeholders and local environmental conditions (Van Holt et al., 2012). Our results therefore could have important applications for conservation. For instance, the fact that the relative abundances within functional traits change—despite no trait combination is lost or added at ecological scales—can be seen as evidence for imminent functional extinctions and turnover (Boersma et al., 2016; Säterberg, Sellman, & Ebenman, 2013). Accordingly, the analysis of the combined variation of functional richness and dispersion can be a useful tool to prevent the extinction of critical functional traits.

In summary, our results indicate that considering different facets of functional diversity allows the identification of differing spatiotemporal patterns of variation of community assembly processes. Although the mechanisms that generate variation in functional diversity were not experimentally assessed in this study, the well‐known effects of the vertical environmental stress gradient in rocky intertidal habitats support the idea that the interplay between environmental filters and niche complementarity generates a continuum between functional convergence and divergence. Both environmental and anthropogenic factors could be analyzed to explain the significant spatial (horizontal) and temporal variation in the patterns of functional dispersion along the vertical intertidal environmental gradient. Future work on this issue could help to disentangle the impact of human disturbances on the functional structure of aquatic and terrestrial communities worldwide. For instance, a trait‐based approach would allow us to assess anthropogenic impacts across biogeographic regions with different levels of taxonomic and phylogenetic diversity. The integrated analysis of different facets of functional diversity can provide useful information about the variation and potential for local extinction of functional traits that ultimately influence ecosystem functioning.

## Conflict of Interest

None declared.

## References

[ece32762-bib-0001] Aguilera, M. A. , & Navarrete, S. A. (2012). Functional identity and functional structure change through succession in a rocky intertidal marine herbivore assemblage. Ecology, 93, 75–89. 22486089 10.1890/11-0434.1

[ece32762-bib-1000] Ancapichún S. , & Garcés‐Vargas J. (2015) Variability of the Southeast Pacific Subtropical Anticyclone and its impact on sea surface temperature off north‐central Chile. Ciencias Marinas 41, 1‐20.

[ece32762-bib-0002] Anderson, M. J. , Ellingsen, K. E. , & McArdle, B. H. (2006). Multivariate dispersion as a measure of beta diversity. Ecology Letters, 9, 683–693. 16706913 10.1111/j.1461-0248.2006.00926.x

[ece32762-bib-0003] Berthelsen, A. K. , Hewitt, J. E. , & Taylor, R. B. (2015). Biological traits and taxonomic composition of invertebrate assemblages associated with coralline turf along an environmental gradient. Marine Ecology Progress Series, 530, 15–27.

[ece32762-bib-0004] Boaventura, D. , Alexander, M. , Della Santina, P. , Smith, N. D. , Re, P. , da Fonseca, L. C. , & Hawkins, S. J. (2002). The effects of grazing on the distribution and composition of low‐shore algal communities on the central coast of Portugal and on the southern coast of Britain. Journal of Experimental Marine Biology and Ecology, 267, 185–206.

[ece32762-bib-0005] Boersma, K. S. , Dee, L. E. , Miller, S. J. , Bogan, M. T. , Lytle, D. A. , & Gitelman, A. I. (2016). Linking multidimensional functional diversity to quantitative methods: A graphical hypothesis‐evaluation framework. Ecology, 97, 583–593. 27197386 10.1890/15-0688

[ece32762-bib-0006] Broitman, B. R. , Véliz, F. , Manzur, T. , Wieters, E. A. , Finke, G. R. , Fornes, P. A. , … Navarrete, S. A. (2011). Geographic variation in diversity of wave exposed rocky intertidal communities along central Chile. Revista Chilena de Historia Natural, 84, 143–154.

[ece32762-bib-0007] Brusca, R. C. , & Brusca, G. J. (2003). Invertebrates. Sunderland, MA: Sinauer Associates.

[ece32762-bib-0008] Bulleri, F. , Benedetti‐Cecchi, L. , Cusson, M. , Maggi, E. , Arenas, F. , Aspden, R. , … Paterson, D. M. (2012). Temporal stability of European rocky shore assemblages: Variation across a latitudinal gradient and the role of habitat‐formers. Oikos, 121, 1801–1809.

[ece32762-bib-0009] Burnham, K. P. , & Anderson, D. R. (2004). Multimodel inference—Understanding AIC and BIC in model selection. Sociological Methods & Research, 33, 261–304.

[ece32762-bib-0010] Carmona, C. P. , de Bello, F. , Mason, N. W. H. , & Leps, J. (2016). Traits without borders: Integrating functional diversity across scales. Trends in Ecology & Evolution, 31, 382–394. 26924737 10.1016/j.tree.2016.02.003

[ece32762-bib-0011] Caro, A. U. , Navarrete, S. A. , & Castilla, J. C. (2010). Ecological convergence in a rocky intertidal shore metacommunity despite high spatial variability in recruitment regimes. Proceedings of the National Academy of Sciences of the United States of America, 107, 18528–18532. 20937867 10.1073/pnas.1007077107 PMC2972975

[ece32762-bib-0012] Cavanaugh, K. C. , Siegel, D. A. , Reed, D. C. , & Dennison, P. E. (2011). Environmental controls of giant‐kelp biomass in the Santa Barbara Channel, California. Marine Ecology Progress Series, 429, 1–17.

[ece32762-bib-0013] Chen, C. L. A. , Soong, K. , & Chen, C. A. (2008). The smallest oocytes among broadcast‐spawning actiniarians and a unique lunar reproductive cycle in a unisexual population of the sea anemone, *Aiptasia pulchella* (Anthozoa: Actiniaria). Zoological Studies, 47, 37–45.

[ece32762-bib-0014] Connell, J. H. (1961). The influence of interspecific competition and other factors on the distribution of the barnacle *Chathamalus stellatus* . Ecology, 42, 710–723.

[ece32762-bib-0015] de Bello, F. , Leps, J. , & Sebastia, M. T. (2006). Variations in species and functional plant diversity along climatic and grazing gradients. Ecography, 29, 801–810.

[ece32762-bib-0016] Donahue, M. J. , Desharnais, R. A. , Robles, C. D. , & Arriola, P. (2011). Mussel bed boundaries as dynamic equilibria: Thresholds, phase shifts, and alternative states. American Naturalist, 178, 612–625. 10.1086/662177 22030731

[ece32762-bib-0017] Fukami, T. , Bezemer, T. M. , Mortimer, S. R. , & van der Putten, W. H. (2005). Species divergence and trait convergence in experimental plant community assembly. Ecology Letters, 8, 1283–1290.

[ece32762-bib-0018] Gelcich, S. , Hughes, T. P. , Olsson, P. , Folke, C. , Defeo, O. , Fernández, M. , … Castilla, J. C. (2010). Navigating transformations in governance of Chilean marine coastal resources. Proceedings of the National Academy of Sciences of the United States of America, 107, 16794–16799. 20837530 10.1073/pnas.1012021107 PMC2947917

[ece32762-bib-0019] Gómez, I. , & Huovinen, P. (2011). Morpho‐functional patterns and zonation of South Chilean seaweeds: The importance of photosynthetic and bio‐optical traits. Marine Ecology‐Progress Series, 422, 77–91.

[ece32762-bib-0020] Gotelli, N. J. , & McCabe, D. J. (2002). Species co‐occurrence: A meta‐analysis of J. M. Diamond's assembly rules model. Ecology, 83, 2091–2096.

[ece32762-bib-0021] Griffin, J. N. , Mendez, V. , Johnson, A. F. , Jenkins, S. R. , & Foggo, A. (2009). Functional diversity predicts overyielding effect of species combination on primary productivity. Oikos, 118, 37–44.

[ece32762-bib-0023] Harley, C. D. G. , & Helmuth, B. S. T. (2003). Local‐ and regional‐scale effects of wave exposure, thermal stress, and absolute versus effective shore level on patterns of intertidal zonation. Limnology and Oceanography, 48, 1498–1508.

[ece32762-bib-0024] Hawkins, S. J. , & Hartnoll, R. G. (1983). Grazing of intertidal algae by marine invertebrates. Oceanography and Marine Biology 21, 195‐282.

[ece32762-bib-0025] Hines, A. H. (1986). Larval patterns in the life histories of Brachyuran crabs (Crustacea, Decapoda, Brachyura). Bulletin of Marine Science, 39, 444–466.

[ece32762-bib-0026] Hooper, D. U. , Chapin, F. S. , Ewel, J. J. , Hector, A. , Inchausti, P. , Lavorel, S. , … Wardle, D. A. (2005). Effects of biodiversity on ecosystem functioning: A consensus of current knowledge. Ecological Monographs, 75, 3–35.

[ece32762-bib-0027] Jara, H. F. , & Moreno, C. A. (1984). Herbivory and structure in a midlittoral rocky community: A case in southern Chile. Ecology, 65, 28–38.

[ece32762-bib-0028] Kay, M. C. , & Emlet, R. B. (2002). Laboratory spawning, larval development, and metamorphosis of the limpets *Lottia digitalis* and *Lottia asmi* (Patellogastropoda, Lottiidae). Invertebrate Biology, 121, 11–24.

[ece32762-bib-0029] Lagos, N. A. , Castilla, J. C. , & Broitman, B. R. (2008). Spatial environmental correlates of benthic recruitment: A test using intertidal barnacles along the coast of northern Chile. Ecological Monographs, 78, 245–261.

[ece32762-bib-0030] Laliberté, E. , & Legendre, P. (2010). A distance‐based framework for measuring functional diversity from multiple traits. Ecology, 91, 299–305. 20380219 10.1890/08-2244.1

[ece32762-bib-0031] Leibold, M. A. , Holyoak, M. , Mouquet, N. , Amarasekare, P. , Chase, J. M. , Hoopes, M. F. , … Gonzalez, A. (2004). The metacommunity concept: A framework for multi‐scale community ecology. Ecology Letters, 7, 601–613.

[ece32762-bib-0032] Letelier, J. , Pizarro, O. , & Nunez, S. (2009). Seasonal variability of coastal upwelling and the upwelling front off central Chile. Journal of Geophysical Research‐Oceans, 114, 1–16.

[ece32762-bib-0033] Levin, L. A. , & Bridges, T. S. (1995). Patterns and diversity in reproduction and development In McEdward L. R. (Ed.), Ecology of marine invertebrate larvae (pp. 1–48). Boca Raton, FL: CRC Press.

[ece32762-bib-0034] MacArthur, R. , & Levins, R. (1967). The limiting similarity, convergence, and divergence of coexisting species. The American Naturalist, 101, 377–385.

[ece32762-bib-0035] Manly, B. , & Sanderson, J. G. (2002). A note on null models: Justifying the methodology. Ecology, 83, 580–582.

[ece32762-bib-0036] Mason, N. W. H. , de Bello, F. , Mouillot, D. , Pavoine, S. , & Dray, S. (2013). A guide for using functional diversity indices to reveal changes in assembly processes along ecological gradients. Journal of Vegetation Science, 24, 794–806.

[ece32762-bib-0037] Mason, N. W. H. , Lanoiselee, C. , Mouillot, D. , Wilson, J. B. , & Argillier, C. (2008). Does niche overlap control relative abundance in French lacustrine fish communities? A new method incorporating functional traits. Journal of Animal Ecology, 77, 661–669. 18397248 10.1111/j.1365-2656.2008.01379.x

[ece32762-bib-0038] Mason, N. W. H. , Richardson, S. J. , Peltzer, D. A. , de Bello, F. , Wardle, D. A. , & Allen, R. B. (2012). Changes in coexistence mechanisms along a long‐term soil chronosequence revealed by functional trait diversity. Journal of Ecology, 100, 678–689.

[ece32762-bib-0039] Mason, N. W. H. , Wiser, S. K. , Richardson, S. J. , Thorsen, M. J. , Holdaway, R. J. , Dray, S. , … Carswell, F. E. (2013). Functional traits reveal processes driving natural afforestation at large spatial scales. PLoS One, 8, e75219. 24058664 10.1371/journal.pone.0075219 PMC3776731

[ece32762-bib-0040] Menge, B. A. , & Branch, G. M. (2001). Rocky intertidal communities In Bertness M. D., Gaines S. D., & Hay M. E. (Eds.), Marine community ecology (pp. 221–251). Sunderland, MA: Sinauer Associates.

[ece32762-bib-0041] Menge, B. A. , Gouhier, T. C. , Hacker, S. D. , Chan, F. , & Nielsen, K. J. (2015). Are meta‐ecosystems organized hierarchically? A model and test in rocky intertidal habitats. Ecological Monographs, 85, 213–233.

[ece32762-bib-0042] Menge, B. A. , & Menge, D. N. L. (2013). Dynamics of coastal meta‐ecosystems: The intermittent upwelling hypothesis and a test in rocky intertidal regions. Ecological Monographs, 83, 283–310.

[ece32762-bib-0043] Moran, A. L. (1997). Spawning and larval development of the black turban snail *Tegula funebralis* (Prosobranchia: Trochidae). Marine Biology, 128, 107–114.

[ece32762-bib-0044] Moreno, C. A. (2001). Community patterns generated by human harvesting on Chilean shores: A review. Aquatic Conservation‐Marine and Freshwater Ecosystems, 11, 19–30.

[ece32762-bib-0045] Moreno, C. A. , & Jaramillo, E. (1983). The role of grazers in the zonation of intertidal macroalgae of the Chilean coast. Oikos, 41, 73–76.

[ece32762-bib-0046] Morgan, S. G. , & Fisher, J. L. (2010). Larval behavior regulates nearshore retention and offshore migration in an upwelling shadow and along the open coast. Marine Ecology Progress Series, 404, 109–126.

[ece32762-bib-0047] Mouchet, M. A. , Villéger, S. , Mason, N. W. H. , & Mouillot, D. (2010). Functional diversity measures: An overview of their redundancy and their ability to discriminate community assembly rules. Functional Ecology, 24, 867–876.

[ece32762-bib-0048] Mudrak, O. , Janecek, S. , Gotzenberger, L. , Mason, N. W. H. , Hornik, J. , de Castro, I. , … de Bello, F. (2016). Fine‐scale coexistence patterns along a productivity gradient in wet meadows: Shifts from trait convergence to divergence. Ecography, 39, 338–348.

[ece32762-bib-0049] Narvaez, D. A. , Navarrete, S. A. , Largier, J. , & Vargas, C. A. (2006). Onshore advection of warm water, larval invertebrate settlement, and relaxation of upwelling off central Chile. Marine Ecology Progress Series, 309, 159–173.

[ece32762-bib-0050] Navarrete, S. A. , & Castilla, J. C. (2003). Experimental determination of predation intensity in an intertidal predator guild: Dominant versus subordinate prey. Oikos, 100, 251–262.

[ece32762-bib-0051] Navarrete, S. A. , Wieters, E. A. , Broitman, B. R. , & Castilla, J. C. (2005). Scales of benthic‐pelagic coupling and the intensity of species interactions: From recruitment limitation to top‐down control. Proceedings of the National Academy of Sciences of the United States of America, 102, 18046–18051. 16332959 10.1073/pnas.0509119102 PMC1312419

[ece32762-bib-0052] Nielsen, K. J. , & Navarrete, S. A. (2004). Mesoscale regulation comes from the bottom‐up: Intertidal interactions between consumers and upwelling. Ecology Letters, 7, 31–41.

[ece32762-bib-0053] Paine, R. (1966). Food web complexity and species diversity. American Naturalist, 100, 65–75.

[ece32762-bib-0054] Paine, R. T. (1974). Intertidal community structure. Oecologia, 15, 93–120. 10.1007/BF00345739 28308255

[ece32762-bib-0055] Pavoine, S. , & Bonsall, M. B. (2011). Measuring biodiversity to explain community assembly: A unified approach. Biological Reviews, 86, 792–812. 21155964 10.1111/j.1469-185X.2010.00171.x

[ece32762-bib-0056] Petchey, O. L. , & Gaston, K. J. (2006). Functional diversity: Back to basics and looking forward. Ecology Letters, 9, 741–758. 16706917 10.1111/j.1461-0248.2006.00924.x

[ece32762-bib-0057] Pinochet‐Velásquez, A. S. (2015). Variación temporal de la surgencia en la región centro‐sur de Chile (39º‐41º) a través de información procedente de satélites. Valdivia, Chile: Facultad de Ciencias.

[ece32762-bib-0058] Piñones, A. , Castilla, J. C. , Guiñez, R. , & Largier, J. L. (2007). Nearshore surface temperatures in Antofagasta Bay (Chile) and adjacent upwelling centers. Ciencias Marinas, 33, 37–48.

[ece32762-bib-0059] R Core Team (2016). R: A language and environment for statistical computing. Vienna, Austria: R Foundation for Statistical Computing.

[ece32762-bib-0060] Raffaelli, D. , & Hawkins, S. (1996). Intertidal ecology (356 pp.). London, UK: Chapman & Hall.

[ece32762-bib-0061] Reynoso‐Granados, T. , Monsalvo‐Spencer, P. , Serviere‐Zaragoza, E. , & Del Proo, S. A. G. (2007). Larval and early juvenile development of the volcano keyhole limpet, *Fissurella volcano* . Journal of Shellfish Research, 26, 65–70.

[ece32762-bib-0022] Ríos, C. C. , Noziglia, N. C. , Guzmán, M. L. (1987). Larval development of the gastropods *Siphonaria lessoni* blainville 1824 and *Kerguelenella lateralis* Gould 1946 *Pulmonata siphonariidae* from the strait of Magellan Chile. Anales del Instituto de La Patagonia Serie Ciencias Naturales, 17, 77–88.

[ece32762-bib-0062] Roughgarden, J. , Gaines, S. , & Possingham, H. (1988). Recruitment dynamics in complex life cycles. Science, 241, 1460–1466. 11538249 10.1126/science.11538249

[ece32762-bib-0063] Saelzer, H. E. , Quintana, R. , & Quiñones, R. (1986). Larval development of *Petrolisthes granulosus* (Guérin, 1835) (Decapoda: Anomura: Porcellanidae) under Laboratory conditions. Journal of Crustacean Biology, 6, 804–819.

[ece32762-bib-0064] Säterberg, T. , Sellman, S. , & Ebenman, B. (2013). High frequency of functional extinctions in ecological networks. Nature, 499, 468–470. 23831648 10.1038/nature12277

[ece32762-bib-0065] Shinen, J. L. , & Navarrete, S. A. (2010). Coexistence and intertidal zonation of chthamalid barnacles along central Chile: Interference competition or a lottery for space? Journal of Experimental Marine Biology and Ecology, 392, 176–187.

[ece32762-bib-0066] Shinen, J. L. , & Navarrete, S. A. (2014). Lottery coexistence on rocky shores: Weak niche differentiation or equal competitors engaged in neutral dynamics? The American Naturalist, 183, 342–362. 10.1086/674898 24561598

[ece32762-bib-0067] Somero, G. N. (2010). The physiology of climate change: How potentials for acclimatization and genetic adaptation will determine ‘winners’ and ‘losers’. Journal of Experimental Biology, 213, 912–920. 20190116 10.1242/jeb.037473

[ece32762-bib-0068] Stephenson, T. A. , & Stephenson, A. (1949). The universal features of zonation between tide‐marks on rocky coasts. Journal of Ecology, 37, 289–305.

[ece32762-bib-0069] Tejada‐Martinez, D. , López, D. N. , Bonta, C. C. , Sepúlveda, R. D. , & Valdivia, N. (2016). Positive and negative effects of mesograzers on early‐colonizing species in an intertidal rocky‐shore community. Ecology and Evolution, 6, 2045–7758. 10.1002/ece3.2323 PMC4983589 27547352

[ece32762-bib-0070] Underwood, A. J. , & Jernakoff, P. (1981). Effects of interactions between algae and grazing gastropods on the structure of a low‐shore inter‐tidal algal community. Oecologia, 48, 221–233. 10.1007/BF00347968 28309804

[ece32762-bib-0071] Underwood, A. J. , & Jernakoff, P. (1984). The effects of tidal height, wave‐exposure, seasonality and rock‐pools on grazing and the distribution of intertidal macroalgae in New‐South‐Wales. Journal of Experimental Marine Biology and Ecology, 75, 71–96.

[ece32762-bib-0072] Valdivia, N. , Aguilera, M. A. , Navarrete, S. A. , & Broitman, B. R. (2015). Disentangling the effects of propagule supply and environmental filtering on the spatial structure of a rocky shore metacommunity. Marine Ecology Progress Series, 538, 67–79.

[ece32762-bib-0073] Valdivia, N. , de la Haye, K. L. , Jenkins, S. R. , Kimmance, S. A. , Thompson, R. C. , & Molis, M. (2009). Functional composition, but not richness, affected the performance of sessile suspension‐feeding assemblages. Journal of Sea Research, 61, 216–221.

[ece32762-bib-0074] Valdivia, N. , Gollety, C. , Migne, A. , Davoult, D. , & Molis, M. (2012). Stressed but stable: Canopy loss decreased species synchrony and metabolic variability in an intertidal hard‐bottom community. PLoS One, 7, e36541. 22574181 10.1371/journal.pone.0036541 PMC3344890

[ece32762-bib-0075] Van Holt, T. , Moreno, C. A. , Binford, M. W. , Portier, K. M. , Mulsow, S. , & Frazer, T. K. (2012). Influence of landscape change on nearshore fisheries in southern Chile. Global Change Biology, 18, 2147–2160.

[ece32762-bib-0076] Venegas, R. M. , Ortiz, V. , Olguin, A. , & Navarrete, S. A. (2000). Larval development of the intertidal barnacles *Jehlius cirratus* and *Notochthamalus scabrosus* (Cirripedia: Chthamalidae) under laboratory conditions. Journal of Crustacean Biology, 20, 495–504.

[ece32762-bib-0077] Villéger, S. , Mason, N. W. H. , & Mouillot, D. (2008). New multidimensional functional diversity indices for a multifaceted framework in functional ecology. Ecology, 89, 2290–2301. 18724739 10.1890/07-1206.1

