# Supplementary material for: Context‐dependent functional dispersion across similar ranges of trait space covered by intertidal rocky shore communities
Source: Ecol Evol. 2017 Feb 17;7(6):1882–91. doi: 10.1002/ece3.2762 (PMC5355202; doi:10.1002/ece3.2762)
Supplement: Supplementary file 1 [file ECE3-7-1882-s001.docx]

*Supplementary material of the article entitled*

**Context-dependent functional dispersion across similar ranges of trait space covered by intertidal rocky shore communities**

Nelson Valdivia^1, 2, *^, Viviana Segovia-Rivera^1^, Eliseo Fica^1^, César C. Bonta^1^, Moisés A. Aguilera^3^, Bernardo R. Broitman^4^

^1^Instituto de Ciencias Marinas y Limnológicas, Facultad de Ciencias, Universidad Austral de Chile, Campus Isla Teja, Valdivia, Chile

^2^Centro FONDAP de Investigación de Dinámicas de Ecosistemas Marinos de Altas Latitudes (IDEAL)

^3^Departamento de Biología Marina, Facultad de Ciencias del Mar, Universidad Católica del Norte, Larrondo 1281, Coquimbo, Chile

^4^Centro de Estudios Avanzados en Zonas Áridas (CEAZA), Universidad Católica del Norte, Larrondo 1281, Coquimbo, Chile

*****Corresponding author. Tel.: +56632221557, Fax: +56632221455, E-mail: nelson.valdivia@uach.cl

Fig. S1. Redundancy analysis ordination plot showing the relationships between sea surface temperature (SST) and chlorophyll-a concentration (Chl-a) at the four sampling sites (CHEU: Cheuque; CALF: Calfuco; CHAI: Chaihuín; PUCA: Pucatrihue). We analysed monthly values (2003 – 2014) of MODIS SST and chlorophyll-a data downloaded from http://oceancolor.gsfc.nasa.gov/.
